# Coordinating Cognition: The Entorhinal Cortex in Mnemonic, Temporal and Spatial Representation

**DOI:** 10.3390/cells15121063

**Published:** 2026-06-10

**Authors:** Sara Marcoccia, Giulia Chiacchierini, Patrizia Campolongo

**Affiliations:** 1 Department of Physiology and Pharmacology, Sapienza University of Rome, 00185 Rome, Italy; 2 Neuropharmacology Unit, IRCCS Santa Lucia Foundation, 00143 Rome, Italy

**Keywords:** entorhinal cortex, MEC, LEC, connectivity, memory, navigation, time processing

## Abstract

**Highlights:**

**What are the main findings?**

- MEC and LEC jointly support episodic, spatial and working memory.
- EC network dysfunction underlies cognitive decline in brain disorders.

**What are the implications of the main findings?**

- Medial (MEC) and lateral entorhinal cortex (LEC) form interacting, not segregated, functional systems.
- The EC is a dynamic system integrating space, time and context for cognition.

**Abstract:**

The entorhinal cortex (EC) is a central structure of the medial temporal lobe, functioning as the main cortical gateway to the hippocampus (HPC) and playing a crucial role in memory, spatial navigation, and temporal representation. This review outlines the distinct yet complementary contributions of its two main subdivisions, the medial (MEC) and lateral (LEC) entorhinal cortices. Despite being historically viewed as functionally segregated, they operate instead in close coordination to support the encoding and retrieval of multidimensional experiences. While the MEC is prominently involved in mapping spatial relationships and movement through specialized cell populations, and the LEC in processing object-related and contextual information, growing evidence shows substantial integration between these domains, challenging strict dichotomies. The MEC encodes elapsed time through persistent firing and time cell sequences, while the LEC signals temporal context via rate remapping; their convergent projections to the hippocampus enable the formation of temporally structured episodic memories. The review assesses recent findings on memory, navigation, and time processing, and highlights how the EC supports each through its layered architecture, local microcircuitry, and widespread interactions with HPC, cortical, and subcortical networks. Moreover, alterations in EC activity patterns emerge as the earliest signs of pathologies such as Alzheimer’s disease and temporal lobe epilepsy. Altogether, this review offers an up-to-date view of the EC not as a set of parallel modules, but as a highly interactive and dynamic system essential for structuring experience across space, time, and context.

## 1. Introduction

The Entorhinal Cortex (EC) is a crucial component of the medial temporal lobe [1,2]. It serves as a critical interface and a central relay station for the main information streams projecting to and from the hippocampal (HPC) formation [3,4]. In addition to its reciprocal connections with the HPC, the EC maintains widespread interactions with neocortical and subcortical regions, positioning it as a hub for integrating multimodal sensory, mnemonic, and contextual information [2,5].

Its pivotal position within these circuits is reflected not only in its contribution to memory and information integration but also in its marked vulnerability to neurological disorders [4,6]. For example, in Alzheimer’s disease (AD), the EC, particularly Layer II, is among the earliest and most severely affected regions, with neuronal loss and astrocytic alterations contributing to hallmark memory impairments [4,7]. Similarly, in Temporal Lobe Epilepsy (TLE), selective degeneration in Layer III of the EC is associated with deficits in declarative memory, and comparable patterns of EC damage and cognitive dysfunction are observed in chronic epilepsy models [6]. These findings highlight the EC not only as a key structure in normal mnemonic function but also as an early and vulnerable target in disease, with its degeneration often presaging widespread network dysfunction.

### 1.1. EC’s Anatomical and Functional Organization

The structure of the EC appears to be phylogenetically conserved across different mammalian species [8,9,10], suggesting a fundamental and vital role [10]. In rodents, it is located in the ventroposterior part of the cortical mantle, bordering areas such as the piriform, periamygdaloid, parasubiculum, perirhinal (PER), and postrhinal (POR) cortices. Specifically, its ventral border in the caudomedial portion is with a thin band of the parasubiculum, interposed between the EC and POR cortices. In primates, it is situated on the ventromedial surface of the rostral temporal lobe, bordering the periamygdaloid, pre/parasubiculum, perirhinal, and parahippocampal cortices [2,10].

Adding to this, the functional organization of the rhinal cortices, including the EC, also demonstrates a highly conserved pattern across mammals [5,10]. Dense connections exist between the EC and the HPC, documented in various species including rats, mice, cats, bats, monkeys, and humans. This conserved connectivity scaffold not only facilitates interactions between these structures but also provides the primary pathway for information exchange between the HPC and the rest of the cortex [10].

Building upon this conserved architecture, a fundamental organizational principle of the EC is its subdivision into two major domains: the medial entorhinal cortex (MEC) and the lateral entorhinal cortex (LEC) [5]. This division reflects both distinct connectivity patterns and functional specializations [2,5]. Comparative studies in rodents and primates have identified homologous regions, the rodent MEC corresponding to the posteromedial EC (pmEC) in humans, and the rodent LEC aligning with the anterolateral EC (alEC). These homologies are supported by similarities in cytoarchitecture, connectivity profiles, and representational functions. In both rodents and primates, the MEC/pmEC is predominantly connected to brain areas involved in spatial and sensory processing, such as the presubiculum, POR cortex, or parahippocampal cortex, while the LEC/alEC receives multimodal input from associative regions including the perirhinal, insular, frontal, and cingulate cortices, suggesting a broader role in processing non-spatial, contextual, and object-related information [11].

**Figure 1:**
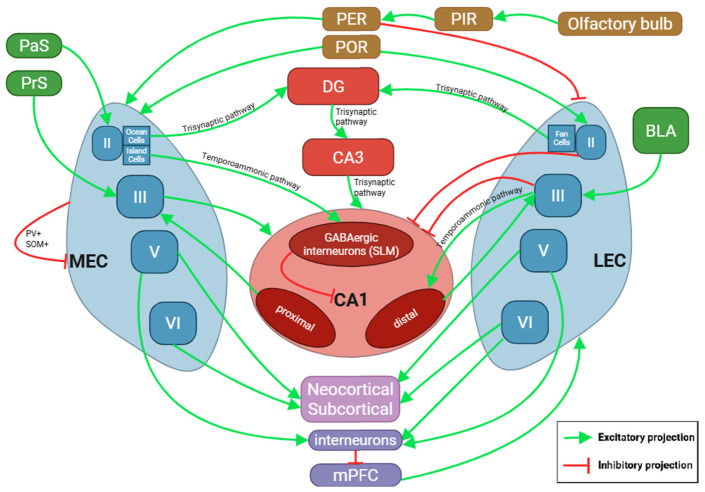
Functional Connectivity of the Entorhinal Cortex. Connectivity map of the entorhinal cortex (EC) and its major cortical and subcortical partners. The schematic highlights the distinct input–output profiles of the medial (MEC) and lateral (LEC) subdivisions. Both MEC and LEC provide major projections to, and receive inputs from, the dentate gyrus (DG) and CA1 subfield of the hippocampus. Both subdivisions also project to neocortical and subcortical targets, as well as to medial prefrontal cortex (mPFC) interneurons, the perirhinal cortex (PER), and the postrhinal cortex (POR). MEC receives prominent inputs from the parasubiculum (PaS) and presubiculum (PrS), while LEC receives direct inputs from the basolateral amygdala (BLA). PV (parvalbumin-expressing interneurons), SOM (somatostatin-expressing interneurons), PIR (piriform cortex), SLM (stratum lacunosum moleculare). Green arrows represent excitatory connections, whereas red arrows represent inhibitory projections. Roman numbers indicate MEC and LEC Layers.

In addition to its mediolateral segmentation, the EC exhibits a layer-specific organization that is also highly conserved across species [10]. Structurally, the EC is composed of six layers, each contributing uniquely to the circuit dynamics [8,10]. The superficial layers, Layers II and III, are primarily efferent, containing principal neurons that send projections via the perforant path to HPC subregions. In contrast, the deep layers, layer V and Layer VI, are primarily afferent, receiving feedback projections from the HPC and relaying outputs to widespread neocortical and subcortical areas (Figure 1) [10,12,13]. These reciprocal projections form closed-loop circuits that enable bidirectional communication between the EC and HPC, a feature considered essential for the temporal coordination and integration of information [14].

Leveraging this anatomical and functional organization, each region supports distinct yet interacting forms of information processing crucial for episodic memory formation, spatial navigation, temporal processing and context-related representation of experience [15,16].

The MEC is most prominently associated with the representation of space and metric navigation [17,18,19]. It hosts a rich diversity of functionally specialized cells, such as grid, head direction (HD), border, speed, and object-vector cells, which together form a sophisticated spatial coding system [17,19]. The most emblematic of these, grid cells, fire in a regular hexagonal pattern across environments, providing a putative internal coordinate system for spatial localization and path integration [20]. These are particularly abundant in MECII, although grid-like activity is also found in MECIII and MECVI, sometimes in conjunctive forms that integrate direction or speed information [21]. The presence of HD cells and border cells, which encode the animal’s orientation or proximity to environmental boundaries, adds geometric constraints to the spatial code [17,21]. Speed cells, modulated by locomotor activity [17,22], and object-vector cells, which anchor firing fields relative to objects [17], further expand the MEC’s coding repertoire, enabling dynamic tracking of self-location relative to both static and movable cues [23].

At the cellular level, MECII is defined by two principal cell types with distinct morphological, molecular, and projection profiles: stellate cells (Reelin-positive (Re^+^), also referred to as ocean cells) and pyramidal cells (Calbindin and Wolfram syndrome 1-positive (Cal/Wsf1^+^), forming clusters also called island cells) [24,25,26]. These subpopulations exhibit segregated organization within MECII, with stellate cells predominantly located between the island cells’ clusters. Stellate cells project robustly to the dentate gyrus (DG), CA3 and CA2 regions, thereby directly feeding the HPC trisynaptic circuit [24,26]. In contrast, Cal/Wsf1^+^ pyramidal cells project to interneurons in the stratum lacunosum-moleculare (SLM) of CA1, exerting feedforward inhibition and thus modulating HPC excitability [26,27]. The microcircuitry within MECII is marked by sparse excitatory connectivity between principal cells and dense inhibitory interactions, primarily mediated by Parvalbumin-positive (PV^+^) cells (Figure 1) [28,29,30]. This inhibitory architecture is thought to be essential for the temporal precision and spatial regularity of grid cell firing [28,30].

Conversely, the LEC is less involved in spatial mapping per se and instead specializes in representing objects, sensory features, and contextual associations [4,5,31]. It plays a key role in item-context binding and in encoding information that is non-metric but critical for episodic memory, such as object identity, familiarity, and temporal order [5,31,32,33]. These representational features stem from its extensive afferents from the perirhinal, insular, frontal, and cingulate cortices, which carry rich multimodal sensory and associative information [22,34,35]. In LECII, the dominant principal cell type is the fan cell [36,37]. They are typically Re^+^ and are known to project robustly to DG, CA3, and CA2, much like MEC stellate cells [33,37]. These cells are suggested to be essential for the integration of object-place-context representations, a function that aligns with the LEC’s role in episodic memory encoding [33,38]. Similar to the MEC, the fan cell network in LEC also exhibits a strong inhibitory motif, involving PV^+^ interneurons and likely supporting temporal coordination during memory tasks [37,39]. However, unlike the MEC, grid-like activity is virtually absent in the LEC, and spatially modulated firing is rare [40,41]. Instead, neurons here tend to exhibit firing that correlates with object presence, identity, and trial-specific features, often without clear spatial tuning [42,43].

Importantly, evidence from human intracranial electrophysiology and functional neuroimaging suggests that many temporal coding mechanisms described in rodents are evolutionarily conserved within the human entorhinal–hippocampal system. High-resolution functional imaging studies have identified a functional organization broadly analogous to the rodent MEC–LEC division, with the pmEC preferentially associated with spatial and contextual processing, and the alEC more strongly linked to object-related and temporal aspects of episodic memory [44,45]. Moreover, intracranial recordings in neurosurgical patients have revealed the presence of “time cells” within both the human hippocampus and EC, characterized by firing patterns selective for specific moments during memory tasks, independently of overt movement or sensory input. Importantly, the stability of these temporal firing sequences predicts the temporal contiguity of memory recall, supporting the idea that temporal coding mechanisms contribute to binding discontinuous events into coherent episodic representations [44,45]. Complementing these findings, ramping cells identified predominantly in the human EC exhibit slowly evolving activity across seconds-to-minutes timescales, potentially providing a broader temporal context for episodic organization. Human recordings also demonstrate preserved theta-phase precession and hippocampal–entorhinal oscillatory synchrony, further supporting the translational relevance of rodent models for understanding temporal representation in the human brain [44].

### 1.2. EC-HPC Connectivity

The EC serves as the major cortical interface for the HPC, through two anatomically and functionally distinct pathways: the indirect trisynaptic pathway and the direct (or temporoammonic) pathway (Figure 1) [26,46].

The trisynaptic pathway is traditionally regarded as the classic route through which EC input reaches the HPC [26]. This pathway originates predominantly from neurons located in MECII and LECII; those projections, called the perforant path, reach the DG, specifically targeting its granule cells, that then project to the CA3 region via mossy fibers, forming the second synapse in the circuit [2,46]. The CA3 pyramidal neurons, in turn, project to the CA1 region through Schaffer collaterals, completing the three-synapse relay that gives this pathway its name [46,47,48]. Within this framework, MECII’s projections [24,26,39] are thought to play a critical role in the processing of spatial and contextual information, providing the HPC with a grid-based metric representation of the environment [18,39], while [39] LEC input may support the encoding of non-spatial contextual features and object-place associations [18,49].

In parallel to this indirect route, the direct or temporoammonic pathway provides a more immediate channel of communication between the EC and the HPC [24,50]. This pathway arises mainly from neurons in MECIII and LECIII and bypasses both the DG and CA3, projecting directly to CA1 pyramidal neurons and the subiculum [24,26,50]. The principal cells involved in the direct pathway are typically pyramidal neurons [51,52], and their role is thought to be particularly important for the formation of temporal associations, such as linking events that occur in sequence [26,52]. This function appears to be especially reliant on input from MECIII [24,50]. Additionally, a complementary regulatory circuit originates from the MECII’s island cells [26,53]. These cells do not directly excite HPC’s pyramidal neurons but instead target interneurons in the stratum lacunosum (SL) of CA1 [26], which in turn provide feed-forward inhibition to CA1 pyramidal cells [24,26]. This inhibition is believed to modulate the excitatory input from MECIII, refining the timing and strength of synaptic responses during temporal learning [24,26,48,53].

On one hand, the trisynaptic circuit is believed to be essential for spatial navigation, contextual memory, pattern separation and pattern completion, processes that enable the HPC to distinguish between similar experiences or retrieve full memories from partial cues [26,54]. On the other hand, the direct pathway plays a key role in temporal sequencing, object-place-context binding, and associative memory formation [5,52,55].

Finally, this bidirectional loop is completed by output projections from CA1 and the subiculum, which return processed information back to the deep layers (V and VI) of the EC [56,57,58]. From there, the EC can redistribute this information to broader cortical areas, thus closing the cortico-HPC loop and allowing for the integration of memory with ongoing perception and behavior [5,58].

This architecture not only underscores the central role of the EC in memory and cognition but also highlights the distinct yet complementary contributions of its medial and lateral subdivisions [5,31,59]: MEC contributing predominantly to spatial and temporal structure, and LEC to object and contextual detail [5,31]. However, growing evidence indicates that the LEC plays a broader role than traditionally appreciated, particularly within domains relevant to memory, spatial navigation, and temporal representation [5,16,55,60,61]. The dual-stream architecture of EC supports the integration of allocentric (external-space-centered) and egocentric (self-centered) information, providing the HPC with the rich multimodal input required to construct flexible and behaviorally relevant episodic memories [17,62,63].

### 1.3. Other Cortical and Subcortical EC’s Connections

Among its most prominent cortical partners, the EC maintains strong bidirectional links with the parahippocampal region, particularly the PER and POR cortices [1,5]. These inputs primarily terminate in superficial layers of the EC, with PER preferentially targeting the LEC and POR more heavily connected to the MEC [1,5,25]. These pathways differentially convey object-related and spatial-contextual information, respectively [63]. The EC also receives substantial input from the presubiculum (PrS) and parasubiculum (PaS), which project mainly to MECII and MECIII, respectively, and are implicated in the transmission of head-directional and spatial signals [22,25,64], thereby contributing to directional coding during navigation [10,65]. Electrical stimulation studies further demonstrate that the dynamic maturation of HD and grid cell properties coincides with developmental changes in PrS–PaS–MEC network ensembles [65]. Interneuronal circuits further refine spatial encoding across EC regions. In the PrS, PV^+^ and somatostatin-positive (SST^+^) interneurons mediate behavioral state-dependent modulation [65,66]. PV^+^ interneurons contribute to disynaptic feedforward inhibition during rapid head turns, tuning directional inputs to the MEC, while SST^+^ cells are implicated in maintaining HD signals during immobility through attractor-like dynamics [66]. Within the MEC itself, PV^+^ and SST^+^ interneurons are known to control distinct space-coding networks, although the specific dynamics of their activity during immobility and fast movements remain to be fully elucidated [25,65].

In addition to parahippocampal structures, the EC is tightly integrated with widespread neocortical areas, especially higher-order polymodal association cortices [5,67]. Notably, the medial prefrontal cortex (mPFC) is reciprocally connected with the LEC, forming a circuit critical for associative recognition memory and object-based episodic processing, while projections from MEC, notably from layer Va, back to mPFC are thought to support remote memory consolidation [24,54,58]. The EC also receives input from visual, parietal, and retrosplenial cortices, with MEC being more heavily targeted by these spatially tuned regions, although the reciprocal connectivity in these circuits is comparatively weak [2,68]. Furthermore, olfactory areas such as the piriform cortex and olfactory bulb provide dense input to the LEC, particularly to its superficial layers, underscoring the LEC’s role in object and odor representation [2,36].

Subcortically, both MEC and LEC maintain substantial connectivity with several key modulatory regions. The amygdala, especially the basolateral subregion (BLA), projects robustly to the EC, particularly LECIII, while EC deep layers send return projections, supporting affective modulation of memory processing [69]. The midline thalamic nuclei, including the nucleus reuniens, send dense projections to multiple EC layers and may serve as a coordinating hub between the mPFC, EC, and HPC [54,70]. Additional subcortical partners include the septum, which provides modulatory gamma-aminobutyric acid (GABA) input to the EC, the claustrum, which targets deep EC layers, and the striatum, which receives output from EC layers IV and V [69,71].

MEC, preferentially layer II, also receives modulatory input from LECII fan cells, which may play a role in contextualizing navigation-related computations [36,38,63]. Fluorescent labeling has revealed that fan cell projections to the MEC and DG exhibit similar intensities, indicating comparable levels of input to both regions. Optogenetic activation of fan cell terminals induces monosynaptic glutamatergic excitation of both stellate and pyramidal neurons in MECII and MECIII, followed by robust feedforward inhibition mediated by GABAergic interneurons [38]. Additionally, LECII fan cells receive direct synaptic inputs from regions such as the piriform cortex, associated with olfaction, and the mPFC, involved in higher-order cognition [36,38]. This connectivity outlines a circuit through which sensory and cognitive signals may influence the MEC, and by extension, HPC processing [38]. This pathway provides a potential anatomical substrate for integrating “what” information from the LEC with the “where” computations of the MEC, especially within its superficial layers. While these findings highlight the convergence of sensory and spatial signals, it remains unclear whether fan cell inputs directly influence the orientation tuning of grid or HD cells [10,38,72].

In summary, the EC emerges as a structurally conserved and functionally versatile hub within the medial temporal lobe, uniquely positioned to mediate the bidirectional flow of information between the HPC and a wide array of cortical and subcortical regions [4]. Its layered and regionally specialized architecture supports the parallel processing of spatial, object-related, contextual, and temporal information, essential capabilities for constructing coherent episodic experiences [4,33]. Moreover, the EC’s early and selective vulnerability in major neurological disorders, such as AD and TLE, underscores its foundational role in cognition and its relevance as a clinical target [4,6]. Building on this anatomical and pathological framework, the following sections will explore in greater detail how the EC contributes to three fundamental cognitive domains: the formation and retrieval of different types of memory (spatial, episodic and working memory), the neural basis of spatial navigation, and the perception and integration of temporal sequences.

## 2. Memory

### 2.1. Spatial Memory

Spatial memory is a fundamental cognitive function that allows organisms to encode, store, and retrieve information about locations and spatial relationships in the environment [73]. The integrity of bidirectional EC-HPC circuit is indispensable for spatial memory, which relies on coordinated activity to generate and maintain coherent cognitive maps [73,74]. Beyond processing object-related information, EC integrates sensory content with contextually relevant spatial information; it provides the HPC with content-rich representations of experience, effectively linking what was encountered with where. Within this network, the EC exerts a critical influence on the content and stability of HPC place representations [75]. Place representations integrate spatial memory and spatial navigation [76]; although this section focuses on memory-related aspects, the role of this system in navigation will be discussed later in Section 3.

#### 2.1.1. LEC’s Role in Spatial Memory

While the MEC has traditionally been considered the primary entorhinal structure involved in spatial memory, the LEC was once thought to play little to no role in this domain [22]. This view was supported by some lesion studies in rodents, which found that damage to the LEC did not impair performance in standard spatial memory and navigation tasks such as the Morris water maze [31,55]. Additionally, early single-unit recordings in open-field environments failed to reveal spatially tuned neurons in the LEC, reinforcing the idea that this region was not involved in encoding spatial information [49,56].

However, subsequent evidence has prompted a substantial re-evaluation of this view [40]. It is now clear that the LEC contributes to spatial memory under specific conditions, particularly when spatial information is tied to discrete stimuli such as objects or environmental context [31,77]. When objects are introduced into the environment, LEC neurons exhibit spatially modulated activity, suggesting that the presence of discrete stimuli can reveal latent spatial coding properties [77,78]. These responses are typically less metrically precise than those observed in the MEC, yet they carry substantial information about object identity, location, and context [42,55]. Although single-unit recordings indicate that LEC neurons are less sensitive to allocentric spatial information than those in the MEC [40], they appear instead to encode space in an egocentric framework, encoding the position of external items relative to the animal rather than within a fixed allocentric coordinate system [42,79]. This egocentric and contextually anchored coding enables the LEC to convey to the HPC a rich, item-specific representation that incorporates perceptual detail and reflects the relative position of salient items [49,55]. Thus, it is considered to play a complementary role to the MEC, particularly in representing the ‘what’ and ‘where’ of external stimuli [49]. Notably, manipulations of LEC activity, such as lesions or targeted inhibition of specific neuronal populations, have been shown to impair object-location and object-context memory, such as novel object recognition in a changed environment or object-context mismatches. In contrast, simple object recognition and pure spatial tasks are largely spared, supporting a role for the LEC in spatial representations tied to the presence and significance of external stimuli [5,54,55,78,80,81].

The LEC receives convergent input from PER and POR cortices, integrating multisensory and associative information into HPC circuits [40]. This afferent connectivity supports its role as a gateway for object-related and contextual signals [54,55]. Signal transfer from PER to LEC occurs with extremely low probability due to strong feedforward inhibition, often referred to as a “wall of inhibition”. This mechanism prevents most incoming information from reaching entorhinal networks unless a coincident input, such as from the amygdala or other cortical regions, is present [82]. In vitro studies support this view, showing that PER stimulation alone rarely activates LEC neurons unless paired with amygdalar input, suggesting that salience signals are crucial to unlock this pathway [43,82]. Consequently, the PER–LEC connection is regarded as discontinuous and selective, highlighting its role in signaling only behaviorally relevant sensory-related changes [40,82]. Moreover, PER–LEC projections also convey strong multisensory integration [55,83], particularly through olfactory drive originating in area 35 of the PER, which receives dense input from the piriform cortex [1], with LEC layer II fan cells being preferentially targeted and showing stronger responsiveness than neighboring pyramidal neurons [36,84,85]. This anatomical organization positions the LEC as critically involved in long-term odor–context associative memory and in the integration of olfactory and tactile stimuli [36,83,84]. These projections further support the integration of sensory and spatial information within EC-HPC circuits [5,81,86].

Although the canonical projections follow the PER–LEC and POR–MEC axes [5,78,87], some degree of overlap exists, as both PER and POR also project to LEC and MEC, albeit to a lesser extent [5,31,62,88]. POR projections to the LEC contribute to its integrative function by providing additional contextual and visuospatial information [5,55,83]. POR activity is associated with the spatial layout of objects and environmental context and may signal changes in the spatial environment [1,82]. Notably, POR–LEC projections mature slightly later than POR–MEC projections in rodents, suggesting that the combined input from POR to both MEC and LEC supports the formation of complex object–place–context associations, which emerge after the initial establishment of spatial navigation functions mediated primarily by the POR–MEC pathway [89].

#### 2.1.2. MEC’s Role in Spatial Memory

The MEC remains the principal actor in the encoding, stabilization, and retrieval of allocentric spatial representations [38,82]. The MEC-HPC network, including the trisynaptic pathway, provides the HPC with spatial and directional cues, which are essential for the establishment of the cognitive map underlying spatial memory formation [82,83]. As such, both lesions [82,84] and chemogenetic activation [90,91,92] studies produce comparable outcomes, namely loss of spatial precision, by provoking enlarged firing fields in CA1 place cells, likely because both chronic lesion and sustained depolarization prevent the MEC from providing a stable, high-fidelity metric reference frame to the HPC, highlighting its role in the accuracy and reliability of HPC spatial coding [82,84,90,91,92,93,94].Transient optogenetic silencing of the MEC induces partial remapping as well, including the recruitment of new fields and suppression of previously active neurons [93] and impairs the temporal organization of CA1 firing including phase precession and theta sequences [95] but do not abolishing overall spatial coding [90,91,93]. This apparent contrast between lesion studies and optogenetic manipulations likely reflects the difference between chronic and transient modulation of MEC input. While permanent lesions remove the primary metric input to the HPC degrading spatial precision, transient inactivation primarily alters map stability and temporal coordination while preserving spatial tuning. Through optogenetics is also possible to disentangle the different contribution of different cell types. As such, MECIII pyramidal neurons and PV^+^ interneurons play distinct roles in maintaining spatial precision and grid cell function [90]. Inactivation of MECIII projections to CA1 reduces spatial precision and increases place field size [92], whereas silencing PV^+^ interneurons disrupts hexagonal grid tuning and speed modulation, highlighting the contribution of inhibitory microcircuits [65,94,96,97]. Overall, these manipulations demonstrate that MEC input is not strictly necessary for the existence of CA1 place fields, but is critical for stabilizing and orienting the active HPC population [65,90,93,94]. Therefore, MEC input ensures accuracy, context specificity, and temporal organization of HPC representations necessary for effective spatial memory [73,90,98].

The concept of remapping, namely the reorganization of HPC spatial representations in response to environmental or internal changes, underscores the dynamic role of the MEC [17,22]. MEC inputs from diverse neuronal populations such as grid, border, and HD cells, which provide the main spatially tuned projections to the HPC, display substantial shifts in their spatial field firing across different environments. This reconfiguration is critical for enabling HPC place cells to encode distinct spatial contexts [75,93]. Remapping events in the MEC might be affected by internal states such as arousal or engagement that may influence the transition between spatial maps [99]. For example, the average running speed during trials where the spatial map changed was significantly slower compared to the average speed in the stable trials. Interestingly, running speed was not found to vary systematically across the different maps within a session [99,100]. This indicates that the correlation is specifically with the transition or event of remapping, rather than a general difference in speed between the states before and after remapping [100].

Within the MECII, stellate cells play a crucial role in spatial memory [101]. Their depolarization induces place cell remapping in CA1 and impair spatial memory performance, even in familiar environments, whereas hyperpolarization alters neuron’s firing without affecting memory or spatial coding. This dissociation highlights that increased, not merely altered, activity in MECII can reconfigure HPC spatial coding [92]. Supporting this, evidence from immediate early genes (IEGs) expression further implicates MECII stellate cells in spatial memory processing. c-Fos expression is elevated in MECII during the early stages of spatial learning, especially in novel environments, reflecting the organism’s learning state [73]. This activity declines with repeated exposure, indicating adaptation (familiar environment). However, in mice with impaired learning, elevated c-Fos expression persists across both familiar and novel contexts, suggesting neuronal hyperactivity and a potential failure in experience-dependent plasticity. Notably, the majority of c-Fos^+^ cells are Re^+^ stellate cells, underscoring their central role in spatial processing and suggesting that dysfunctional plasticity in this population may underlie memory deficits [73,102].

Among the diverse subtypes of MECII stellate cells, a particularly relevant group are the Ocean cells, a population of excitatory stellate neurons in MECII that contribute to spatial memory processing, particularly context representation [27,73,103]. They rapidly form a distinct representation of a novel context, and some maintain a stable response across multiple exposures, integrating sensory information and responding quickly to new contexts by changing their firing rates [103]. Notably the Ocean cells exhibit a distinct speed dependence, similar to grid cells. Given this similarity, it is plausible that Ocean cells might contribute to processing spatial information in relation to movement or environment [103,104].

Beyond layer II, MECIII also contributes to spatial memory, albeit in a different manner [24]. Neurons in layer III predominantly exhibit irregular spatial firing patterns and display weaker theta rhythmicity and HD tuning compared to those in layer II [51,105]. Still, MECIII plays a crucial role in the wider spatial memory circuit. Being the primary source of temporoammonic input to CA1, MECIII is thought to relay spatial and potentially temporal information to the HPC [24,106]. While not forming stereotyped spatial codes like grid cells, MECIII neurons still support the broader spatial memory network and their disruption, as seen in epilepsy models, is associated with spatial memory impairments [51]. Furthermore, in epileptic mice models, the disruption of a distinct population of excitatory neurons in MECIII, specifically a subpopulation of trough-locked neurons that are preferentially active near the trough of theta oscillations, is paralleled by progressive impairments in spatial memory [107]. This dysfunction emerges around eight weeks after pilocarpine-induced epileptic status and is characterized by reduced spike timing precision and weakened phase locking to both local MEC theta and downstream CA1 theta rhythm. These ‘trough-locked’ MECIII neurons project directly to CA1 and are thought to deliver temporally predictive spatial information necessary for phase precession and stable place coding [107].

Collectively, these findings highlight the EC, particularly the MEC, including the distinct contributions of its superficial and deep layers, as a central component of the spatial memory network, supporting the generation and stabilization of hippocampal spatial representations necessary for accurate spatial memory formation and retrieval.

### 2.2. Episodic Memory

**Figure 2:**
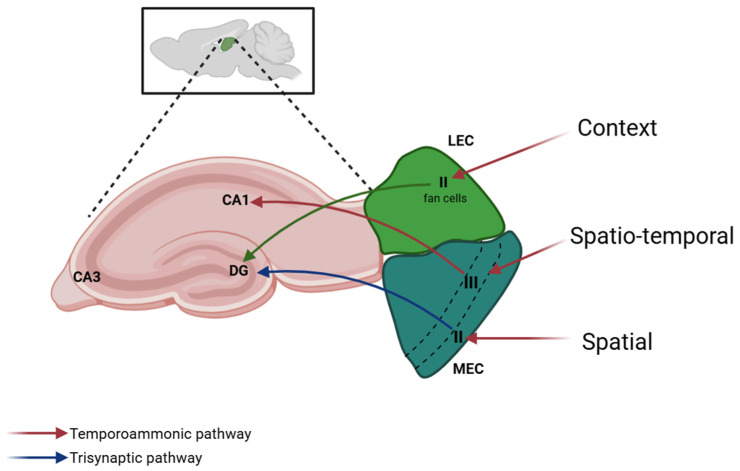
Schematic representation of how MEC (dark green) and LEC (light green) jointly contribute to episodic representation across space, time, and context within the entorhinal–hippocampal network. The EC conveys episodic memory-related information to the hippocampus through complementary MEC and LEC pathways. The MEC primarily provides two complementary streams of information to the hippocampus. The temporoammonic pathway (red arrow), projecting directly from MECIII to CA1, mainly conveys spatio-temporal and temporal sequence information important for temporal association learning and the organization of experience across time. In parallel, the trisynaptic pathway (blue arrow), involving projections from MECII to the DG and CA3 before reaching CA1, predominantly supports allocentric spatial coding, including grid-cell-dependent representations and path integration. LECII fan cells project to the DG (green arrow), providing contextual and object-related information. The convergence of MEC- and LEC-derived signals within hippocampal circuits enables the integration of spatial, temporal, and contextual information into coherent episodic representations. DG: dentate gyrus; CA3: cornu ammonis 3; CA1: cornu ammonis 1; LEC: lateral entorhinal cortex; MEC: medial entorhinal cortex. Dotted lines refer to the zoom-in of the regions of interest. Roman numbers refer to LEC and MEC Layers.

Episodic memory constitutes the system responsible for storing specific personal experiences or events [9]. This type of memory allows for the detailed mental reconstruction of unique events, providing a sense of continuity and identity over time [9,20]. While episodic memory is primarily associated with humans, the concept of “episodic-like memory” is often employed to describe analogous memory processes in non-human animal species [9,20,32]. A distinguishing feature of episodic memory is its capacity to integrate various elements of an experience, including the object identity (what), the spatial location (where), and the temporal context (when) in which the event occurred [9,20]. A critical structure involved is the EC that serves as a vital conduit for relaying information between sensory regions and the HPC, with different regions of the EC playing distinct roles in processing various components of memory [5,16]. In particular, the MEC is proposed to handle spatial information (“where”), while the LEC is primarily involved in processing object-related or non-spatial information (“what”) (Figure 2) [6].

Once again, the LEC was initially regarded as a passive structure with a marginal role in episodic memory; however, accumulating evidence has significantly challenged this view [55,81]. Rats with LECII lesions targeting the fan cells exhibit significant impairment in recognizing new objects compared to both control animals and rats with MEC lesions, suggesting a unique function of the LEC in identifying novel items [31,33,81]. Furthermore, suppression of fan cell activity via synaptic knock-down using the tetanus toxin light chain (TeLC) impairs the ability of mice to discriminate novel object-place-context combinations, while simpler object-context associations remain unaffected [33,108]. The severity of the impairment in episodic-like memory was found to correlate with the number of inactivated fan-cells [33]. Therefore, beyond simple object recognition, LECII neurons respond to both the position of objects within an environment and the specific locations where those objects were encountered [31,54]. These dynamic firing patterns are thought to encode temporal context within an experience, thus linking spatial and temporal aspects of a memory [17,38].

The role of LEC in complex multidimensional memory functions is further highlighted by lesion studies demonstrating impaired associative recognition memory, while sparing recognition of single objects or places [81,83]. Such associative integration of sensory, contextual, and experience-dependent features, rather than precise allocentric spatial computations, is essential for the formation of a cohesive episodic-like memory. Consistently, inhibition of LEC neurons during memory retrieval disrupts episodic-like memory recall, while their stimulation facilitates it [38].

The role of LEC in integrating contextual or temporal information with other types of inputs has been demonstrated also with associative learning tasks. In odor-context association paradigms, the LEC is required to link olfactory cues with contextual information, though not for encoding odors or contexts individually [83]. The LEC has been implicated in olfactory-spatial and olfactory-tactile associative learning, as well as in trace eyeblink conditioning, a temporal associative learning task [109,110]. A powerful tool for probing recognition memory processes under different cognitive demands is the delayed non-match to sample based on odors (DNMO), in which animals are required to identify and select a novel odor over a previously encountered one. In this task, animals are first exposed to a set of odors during a study phase, followed by a delay. During the recognition phase, the same old odors are presented intermixed with novel ones. Rats are trained to withhold digging when encountering an old odor and to dig for a buried reward when presented with a new odor. By varying the number of odors and the length of the delay, the DNMO allows for the manipulation of memory load [111]. In this task deep layers of the LEC are recruited specifically during the retrieval phase under high memory load, implicating this region in non-spatial memory processes that require retrieval mechanisms and the integration of sensory, contextual and temporally associated information [109].

Taken together, these evidences highlight the EC as a central hub for episodic memory, in which spatial (“where”) and non-spatial (“what/when”) information are differentially processed by the MEC and LEC, respectively [5,37]. These complementary computations converge within HPC subregions to form cohesive episodic representations [112]. Importantly, this functional specialization emerges from the EC’s distinct patterns of connectivity with upstream cortical areas such as the PER and POR cortices, and with downstream HPC and prefrontal targets [111]. The next sections will therefore examine these connections in detail, emphasizing how they support the encoding and retrieval of episodic-like memories.

#### 2.2.1. EC-HPC Pathways in Episodic Memory

The HPC represents the core structure for episodic memory, providing the neural substrate for binding together the spatial, temporal, and contextual components of experience into coherent representations [20,43,113]. Its intrinsic organization along the proximodistal axis of CA1, together with dynamic oscillatory interactions with the EC, underlies its ability to segregate and recombine spatial and non-spatial information [20,59,114]. In this section, we review how HPC circuits contribute to episodic memory, emphasizing the role of EC inputs, HPC subfield specialization, and the temporal dynamics that orchestrate memory processing.

Differential connectivity between the MEC and LEC also contributes to the specialized functions of the HPC in memory encoding [60]. The proximal region of CA1 (pCA1) preferentially receives input from the MECIII, which is associated with spatial information processing (“where”), while the distal region of CA1 (dCA1) preferentially receives input from the LECIII, which is more involved in non-spatial, object-related processing [112]. This organization is reflected in the distinct properties of place cells along the transverse axis of CA1 [40,112]. Recent studies using IEG expression markers, such as Zif-268, have shown enhanced expression and cluster-type organization in both the dCA1 and pCA1 regions, corresponding to object and place exposures, respectively [112]. Genetic silencing of MECIII inputs to pCA1 has been shown to induce impairments in temporal association memory [26]. Additionally, MECII Cal/Wsf1^+^ pyramidal neurons project to GABAergic interneurons in CA1, establishing an inhibitory control mechanism that regulates MECIII input to pyramidal neurons, further supporting precise temporal coding of neuron’s firing [24,26]. These findings suggest that the HPC encodes spatial and non-spatial information in a cluster-type organization, segregating these inputs along the CA1 proximodistal axis and mediated by differential EC inputs (Figure 2) [112,115]. Further supporting this functional segregation, direct optogenetic stimulation of the MECIII or LECIII can replicate this cluster-type organization of MECIII-pCA1 and LECIII-dCA1 [112]. Despite clear functional segregation between object-dCA1 and spatial-pCA1, one study observed that spatial information in dCA1 could be higher than in pCA1 when objects were present, suggesting that the presence of objects may influence the spatial encoding properties of CA1 [59,80]. The recognition of LEC neurons in spatial tuning and their encoding of both allocentric and egocentric information related to external items suggest that their projections to dCA1 might contribute to representations in dCA1 that are not exclusively non-spatial, but integrate various aspects of items, context, and possibly time within a spatial framework, contributing to the richness of episodic memory [39,81].

The interaction between gamma (40–100 Hz) and theta oscillations (4–12 Hz), known as theta-gamma coupling, is thought to play an important role in learning item-context associations, which are foundational to episodic memory [116]. During such learning, theta-gamma coupling increases, reflecting the HPC and EC’s coordinated activity in encoding and retrieving episodic-like memories [110,116]. Optogenetically perturbing gamma spike timing in the MEC and LEC resulted in impairments in the encoding phase of spatial and object learning tasks, respectively [112]. This highlights that the precise timing of neuronal activity within gamma cycles in the EC is functionally significant for learning the components of episodic memory (spatial and object information). The impairments in gamma oscillations in epileptogenic rats have been linked to cognitive deficits, further emphasizing their importance in memory retrieval processes [116,117].

Within the EC-HPC circuit, the cooperation between gamma inputs from the EC and CA3 regulates the spike timing of HPC neurons [118]. Specifically, long-range GABAergic projections from ECIII modulate distal dendrites in the SLM of CA1, with precisely timed disinhibition playing a key role in contextual and object memory associations [118,119]. This disinhibition facilitates the formation and retrieval of memories by ensuring that both spatial (MEC) and non-spatial (LEC) inputs are integrated within the HPC to form cohesive episodic memories [39,59]. The presence of long-range inhibition between LEC and MEC inputs to the HPC highlights the importance of coordinated interactions in encoding multimodal memory representations [118,119].

Taken together, these findings highlight that episodic memory relies on the continuous interplay between the EC and the HPC. Through complementary inputs from the MEC and LEC, plasticity at EC-HPC synapses, and the temporal coordination provided by oscillatory dynamics, the EC shapes HPC computations that bind spatial, temporal, and non-spatial features into unified memory traces. This reciprocal dialogue positions the EC-HPC circuit as the fundamental substrate for constructing and retrieving episodic representations.

#### 2.2.2. Other EC Connections Involved in Episodic Memory

The EC is not only a gateway to the HPC but also a critical node in broader cortico-limbic networks that sustain episodic memory. Through reciprocal interactions with the amygdala, prefrontal cortex, and parahippocampal regions, the EC contributes to the integration of episodic traces, linking sensory and spatial inputs with affective salience and higher-order executive processes [4,6,120].

One example of integrating stimuli and context during memory encoding is the CA1-MECV-BLA pathway, playing a key role in generating fear memory engrams during associative learning, such as contextual fear conditioning (CFC), where the animal is required to associate a specific context with an aversive unconditioned stimulus, typically a foot shock [5]. Inhibition of CA1 pyramidal projections to MECV during training disrupts long-term memory formation and impairs downstream consolidation processes in the BLA [52,53]. The EC contributes to the tight dynamic balance between HPC and mPFC engrams during CFC; in fact, while immediate HPC engram formation supports recent memory retrieval, silent engrams in the mPFC gradually mature to an active state and take over, to process remote memory recall [52]. Within this interplay, the EC transmits integrated spatial and non-spatial inputs to the HPC with inhibitory projections, modulating the strength and specificity of these signals, thereby influencing both the formation of active HPC engrams and the emergence of silent mPFC traces [5,121]. Lesions of the EC impair CFC and disrupt fear conditioning to background contextual cues [5]. Pharmacological interference on this pathway, such as muscarinic receptor antagonism, significantly impairs memory retrieval emphasizing the EC’s importance in contextual fear memory [5,122]. Both the MEC and LEC contribute to the contextual aspect of fear memory; in the MEC specifically, muscarinic receptor activation supports persistent firing in principal neurons, particularly in layers III and V. This sustained activity is believed to maintain memory traces over short delays. Thus, muscarinic receptor signaling is essential not only for successful memory retrieval but also for maintaining active memory representations over time [107,122].

The functional interaction of LEC and mPFC is well-established in the context of episodic memory, as disconnection of the mPFC and LEC across hemispheres has been demonstrated to impair associative memory for object identity, spatial location, and contextual information without disrupting memory for these individual components in isolation, as shown instead by disconnection of mPFC-CA3 [5,113]. This suggests a key role for the LEC in maintaining stable representations of learned associations and facilitating memory recall [54,123].

In the broader context of memory processing, the PER and POR cortices contribute significantly to the “what” and “where” streams of information, respectively [79,111]. The PER projects predominantly to the LEC, and its lesions induce deficits in recognition memory, particularly in tasks involving object recognition. On the other hand, the POR, which processes polymodal spatial information, sends excitatory projections to the MEC, contributing to the spatial navigational network [1,78]. The traditional model suggests that the “what” (PER → LEC) and “where” (POR → MEC) streams of information remain largely segregated before converging in the HPC, where they are integrated in regions like the DG and CA3 [18,63]. However, recent research indicates that integration of spatial and non-spatial signals may begin within the EC itself, before reaching the HPC [39].

Taken together, these findings emphasize that the EC supports episodic memory not only through its gateway role to the HPC but also via its broader cortical and subcortical connections. By integrating inputs from the PER and POR, coordinating with the mPFC, and engaging with the BLA, the EC stabilizes associative representations that link spatial, non-spatial, contextual, and emotional information. These interactions allow the EC to modulate both the formation and retrieval of memory traces, maintaining the accuracy and salience of episodic and associative memories across time.

### 2.3. Working Memory

Working memory (WM) refers to a cognitive system responsible for the temporary storage and active manipulation of information essential for ongoing decision-making and cognitive processes. It is characterized by the maintenance of memory for short durations, ranging from milliseconds to tens of seconds. Successful WM performance depends on the coordination and communication between several brain regions [124]. While traditionally associated with mPFC function, substantial evidence implicates the medial temporal lobe, particularly the EC, in WM processes [125].

The EC, particularly the MEC, is engaged in tasks requiring the active retention of information, including spatial WM, delayed match-to-sample, and trace conditioning [3,117]. In particular, persistent firing in MECIII neurons, modulated by cholinergic input, is believed to support this active maintenance, with persistent firing contributing to the retention of spatial and temporal information during WM and temporal association memory tasks [106,126]. These neurons project directly to CA1, where transient bursts of high-frequency gamma synchronization within the EC-HPC circuit, particularly during the maintenance (delay) phase of memory tasks, are thought to enable the temporary retention of information [126]. Notably, optogenetic inhibition of this circuit during the delay phase selectively impairs WM, whereas the same inhibition applied during the encoding or retrieval phases does not significantly affect performance in the WM-guided behavioral tasks, such as the Delayed Matching-to-Place (DMP) or Delayed Non-Match to Place (DNMP) tasks [117,126]. The DMP task is a variant of the Morris Water Maze, in which the hidden platform changes location each day. Animals must encode and retain the platform’s position over a short delay and recall it during a subsequent trial, testing spatial WM rather than long-term reference memory. In contrast, the DNMP task is typically performed in a T-maze or similar apparatus, where animals must remember the location visited in a previous trial and choose the alternate arm after a brief delay, differing from a standard T-maze by the temporal association component rather than simple spatial alternation. These findings indicate that EC-HPC circuit is crucial for the temporary maintenance of information during the ongoing trial, but not for the initial encoding or subsequent retrieval of information [50,66,106,117].

Another specific circuit of interest in WM involves the PaS-EC pathway, which is essential during the maintenance phase of spatial WM [66]. Optogenetic inhibition of PaS-EC terminals impairs WM performance specifically during the delay phase, but not the retrieval, of the DNMP task, highlighting the critical involvement of this circuit in the temporary storage and retention of information necessary for WM [127]. The precise mechanisms by which this pathway supports WM maintenance are an area of ongoing investigation. One proposed mechanism suggests that the anteroventral subdivision of the anterior thalamic nuclei, which in turn projects to the PaS, may be involved in contributing to the generation of high-frequency gamma oscillations observed in the EC during the delay period, which have been correlated with successful WM performance [26,66,128].

Finally, the septal projections to the MEC are crucial for WM regulation. In particular, the septal projections from PV^+^ and Cal/Wsf1^+^ cells modulate the theta rhythmicity and phase precession of MEC neurons [30]. These inhibitory inputs influence the activity of local MEC interneurons and are essential for WM function. Disruption of these projections results in deficits in both context memory and WM, as observed in tasks such as the DMP and DNMP, highlighting the significance of septal modulation in the proper functioning of the MEC [30].

In addition to the MEC’s role in spatial WM, the LEC also exhibits selective engagement during tasks involving non-spatial WM, such as the DNMO task, which requires short-term retention of odor-based information [109,129]. Under high memory load conditions, LEC deep layers are particularly recruited. Studies demonstrate significantly increased activity (e.g., IEG Arc expression) in LEC deep layers when animals are required to retain a larger set of odors, such as 10 odors, compared to lower load conditions like 5 odors [109,130]. In contrast, the superficial layers of the LEC do not show significant engagement in such tasks. This selective, load-dependent activation of deep layers of the LEC suggests that LEC does not act as a buffer for active maintenance, like the persistent firing observed in MEC neurons. Instead, it likely supports associative recall and the integration of mnemonic and contextual cues required for accurate performance under high cognitive demand [4,106,117]. Clinical relevance of EC in WM is noted, particularly in the context of aging and AD [4]. Changes in WM function are considered prodromal features of AD, and tasks assessing spatial navigation, which involve WM, are suggested for early clinical evaluation to detect incipient cognitive decline [103]. These changes reflect specific neural alterations: aging and AD disrupt the EC-HPC circuit through early synaptic loss in ECII, reduced LTP in perforant path [4], and diminished excitability in circuits such as the AV–PaS–EC loop, critical for maintaining spatial WM [66]. In addition, altered neural timing and reduced theta synchrony between MEC and HPC also correlate with early WM deficits. Collectively, these changes impair the ability to form, maintain, and retrieve transient WM traces, and it is an early cognitive marker of AD [102].

To summarize, the EC emerges as a central node in the distributed network supporting WM. Both MEC and LEC contribute in domain-specific ways; MEC primarily to spatial and temporal aspects of memory, and LEC to non-spatial information [3,31]. The ability of MECIII neurons to sustain persistent activity, regulated by cholinergic and septal inputs, underscores their role in active memory maintenance. Moreover, interactions between the EC and HPC, modulated by gamma and theta oscillations, are crucial during the delay phase of WM tasks [105]. Thalamocortical pathways and septal projections further refine this process, reinforcing the EC’s integrative function [66]. Importantly, alterations in EC circuits are linked to pathological conditions such as AD, emphasizing the clinical relevance of this region in the early detection and understanding of memory impairments [4,102]. Altogether, these findings establish the EC not merely as a relay structure, but as an active participant in the encoding, maintenance, and retrieval of information critical to WM.

### 2.4. EC and Memory: Therapeutic Approaches

The therapeutic potential of targeting the EC, particularly spatial memory, is underscored by findings from deep brain stimulation (DBS) rodent studies [131,132], although the translational relevance of these findings remains to be established. Acute EC-DBS, particularly when applied to the MEC, has been shown to enhance spatial memory and stimulate neurogenesis in the DG, within 3–5 days post-stimulation. Importantly, this memory improving effect was abolished when neurogenesis was blocked [133], suggesting that the integration of newborn neurons into HPC circuits is essential for the observed memory improvements [131,133]. Beyond promoting neurogenesis, EC-DBS also induces short-term increases in HPC-mPFC functional connectivity and enhances theta-gamma coupling, both linked to improved spatial discrimination. Functional MRI (fMRI) studies in healthy rats show this increased connectivity three days after EC-DSB. These effects, however, are transient, returning to baseline levels within four weeks, underscoring the dynamic and reversible nature of this neuromodulatory intervention [131].

While neurogenesis is not restricted to the EC, stimulation of EC efferents to the HPC has been shown to produce rapid effects on memory retrieval, including vivid autobiographical recall, indicating additional non-neurogenic contributions to memory facilitation [131,132].

EC-DBS has also been explored in the context of neurodegenerative diseases like AD, where the EC is one of the first regions to exhibit pathological features; preclinical studies suggest that EC-DBS may reduce amyloid plaque load in both the HPC and EC, and may partially contribute to spatial memory rescue [69,134,135].

Beyond DBS, other potential therapeutic approaches to target the MEC comprise optogenetic, pharmacological, and non-invasive strategies aimed at restoring network function and mitigating the pathology associated with spatial memory deficits [135,136,137]. For instance, theta-burst stimulation (TBS) of excitatory MECII neurons (ECIIPN) in AD mouse models has been shown to protect the ECIIPN–CA1(PV^+^) pathway, restoring the excitatory/inhibitory balance in CA1 microcircuits. TBS-induced activation promoted recovery of spatial learning and memory without affecting non-memory-related behaviors, such as locomotor activity and anxiety-related behaviors in the open field task, demonstrating the potential for circuit-specific interventions [135]. Analogously, low-frequency optogenetic stimulation of the BLA–MEC pathway selectively enhances HPC-dependent spatial/contextual memory consolidation and strengthens local field potential (LFP) in both the MEC and dorsal HPC [120].

Similarly, TLE patients often present declarative memory deficits associated with structural damage in EC regions, including significant loss in layer III. Such degeneration likely disrupts the EC’s role in stabilizing episodic associations and contributes to the memory retrieval deficits commonly seen in both aging and neurodegenerative conditions [6,123].

The LEC may serve as a conduit for broadcasting a global salience signal to the HPC, a function that appears increasingly vulnerable to age-related decline [87,123]. The lateral perforant path (LPP), which comprises projections from LECII to the DG [33], exhibits a presynaptic form of long-term potentiation (LTP) that is significantly impaired by eight months of age in both rats and mice [138]. This age-dependent impairment coincides with reduced retrograde endocannabinoid signaling, which is required for LTP induction, and parallels a decline in performance on LPP-dependent episodic memory tasks. These findings provide a mechanistic link between age-related deficits in LEC plasticity and episodic memory deterioration [138].

Given the pivotal role of the EC in AD pathogenesis, reliable biomarkers are essential not only for early detection but also for monitoring therapeutic efficacy in interventions aimed at restoring EC function and preserving spatial memory [139]. For example, optogenetic inhibition of MECII neurons reduces c-Fos expression in DG neurons, while pharmacological blockade of muscarinic receptors with scopolamine disrupts grid-cell spatial tuning and impairs memory retrieval [73,87,112,140,141,142]. Although these manipulations reflect dysfunction rather than therapeutic rescue, they are crucial as they establish the necessity of MEC activity for normal hippocampal function, providing functional biomarkers, such as c-Fos activity and grid-cell tuning, and synaptic/neuromodulatory targets that following interventions could aim to restore or enhance. As such, chronic EC-DBS reduces Aβ and Tau pathology in HPC and cortical regions of AD or global ischemia models, concomitantly enhancing synaptic markers such as synaptophysin in CA1 [134,136,137].

Collectively, these indicators provide a comprehensive framework for highlighting the EC as a viable therapeutic target, with interventions capable of enhancing neurogenesis, restoring oscillatory dynamics, and reducing pathological burden, all converging to possibly improve spatial memory and cognitive function. However, these applications remain largely experimental and are not yet directly translatable into clinically validated therapeutic approaches.

## 3. Spatial Navigation

In addition to its well-established contribution to memory processes, the EC is also critically involved in the neural mechanisms supporting spatial navigation and orientation. Spatial navigation is a fundamental cognitive process that enables organisms to find their way through an environment and orient themselves in space [71]. This ability, which is crucial for everyday functioning, relies on the integration of various sensory inputs, including information about self-motion (idiothetic) and external cues (allothetic) [130,143]. Spatial navigation is supported by two fundamentally distinct, yet highly interactive reference frames: allocentric and egocentric. Allocentric representations are world-centered, relying on cues external to the organism, typically distal landmarks, whereas egocentric representations are self-centered, rooted in internal or proximal information relative to the body [143,144]. The EC serves as a crucial interface between the neocortex and HPC, integrating multimodal sensory information to guide spatial behavior [21,144].

The MEC, in particular, plays a central role in encoding an animal’s position, orientation, and movement, thereby forming a core component of the brain’s navigation circuitry [71]. Among its most well-characterized cell types are grid cells, which fire at multiple locations forming a periodic hexagonal pattern across an environment, providing a metric for allocentric position [111,144,145]. These grid patterns are not solely dependent on external sensory cues; they can persist in darkness and under reduced sensory input, indicating a robust path integration mechanism based on idiothetic information [113]. Nonetheless, grid cells can also be modulated by environmental features such as borders and landmarks, and their firing can be distorted or anchored by local cues [146]. Border cells, which respond to the presence of environmental boundaries, and HD cells, which encode the animal’s directional orientation relative to distal cues, also contribute critically to allocentric spatial coding within the MEC [147]. Despite the local cues-dependent disruption in grid cells, HD cell maps remain stable, indicating a dissociation between directional orientation coding and spatial periodicity under environmental complexity [148]. Over time and with experience, these representations may stabilize into globally coherent maps even in complex environments [71,148]. Interestingly, HD cells also play a role in egocentric navigation, demonstrating flexible anchoring to both visual and self-motion cues, and some grid cells exhibit conjunctive tuning to both position and head direction, further emphasizing the integrative nature of MEC coding [127] and the EC’s capacity to integrate long-term spatial information [71,148].

### 3.1. Grid Cells Dependent Spatial Navigation

Grid cells are defined by three key parameters: scale, orientation, and spatial phase. The scale denotes the distance between neighboring firing fields; orientation refers to the angular alignment of the grid pattern relative to a reference direction; and spatial phase determines the specific spatial offset of the grid relative to the environment [143]. Grid cells with similar scales and orientations but differing spatial phases collectively tile the environment, enabling a population-level code that supports continuous spatial mapping, organized into functionally discrete modules [143,149,150]. Within a given module, neighboring grid cells tend to share common spatial properties, specifically their orientation and spacing (scale) [143,150]. However, the vertex locations (phases) of their firing fields differ, distributing to cover the entire explored space within that module. This implies that the entire surface of the environment can be represented within a local cell ensemble sharing a common grid spacing and orientation [149,151]. A prominent feature of this modular organization is a progressive increase in grid scale and field size from the dorsal to the ventral parts of the MEC [149]. This increase in scale is described as occurring in discrete steps between modules and it suggests that the larger fields in the ventral MEC may correspond to different roles in spatial navigation compared to the smaller, higher-resolution fields in the dorsal MEC [13,152,153]. This fine-tuning of spatial codes in the MEC is further sculpted by interneuron network dynamics. PV^+^ interneurons, critical for generating gamma oscillations, show greater connectivity in dorsal MEC, potentially accounting for the tighter spatial tuning and enhanced inhibition observed in this region [154,155]. Conversely, ventral MEC appears to support broader fields and reduced inhibitory tone, consistent with its lower PV^+^ interneuron connectivity [156]. This organizational difference in inhibitory circuits contributes to the gradient in spatial resolution and oscillatory coherence along the dorsoventral axis [155,156].

The combination of grids with variable scales, either within the EC or downstream in the HPC, potentially provides a high-resolution spatial coordinate system for navigation over large spaces [143]. This theory has an anatomical base, as grid cells form clustered microcircuits, forming local assemblies in which cells with similar spatial phase offsets are located proximally [152]. This non-random anatomical organization supports the predictions of continuous attractor network (CAN) models, in which cells with similar functional properties, particularly those sharing close phase relationships, are hypothesized to be more strongly connected [147,157]. These models posit that excitatory interactions dominate among cells with small spatial phase differences, while inhibitory interactions prevail between those with greater phase separation [147].

The MEC does not operate solely in response to self-motion cues. It also processes allothetic information (external environmental cues such as visual landmarks) which is critical for anchoring the internal map to the external world [15,93]. Visual inputs influence grid cell stability, and removal of visual cues can disrupt their firing patterns, emphasizing the role of landmark information in maintaining spatial accuracy [113,158]. Neurons in the MEC exhibit conjunctive tuning to landmark and self-motion cues, with cue cells showing selective responses to spatial features in the environment [15,158]. The integration of idiothetic and allothetic cues occurs within individual MEC neurons, supporting a multiplexed coding scheme in which internally generated self-motion signals are continuously combined with external spatial landmarks. This integration is essential for generating and updating the cognitive map, and it allows navigation to be flexible and adaptive in complex, changing environments [15,148].

Revisiting familiar locations allows for error correction using external landmarks, a process likely involving HPC interactions that stabilize entorhinal representations. Over time, MEC dynamics, particularly in large or complex arenas, evolved from local, fragmented patterns to cohesive global maps [148]. This process of map refinement becomes evident through the adaptive reorganization of grid activity when new or behaviorally relevant cues are introduced. For example, the introduction of social or object landmarks can induce rate or global remapping of grid cell activity, indicating a modulation of firing rate and field structure based on salience or cognitive relevance [159,160]. Conversely, under cognitively demanding tasks or in environments that elicit compartmentalization, such as T-mazes, grid representations may fragment, reflecting a transient breakdown of global coherence and dynamically balancing local and global representations to support flexible navigation [161,162].

Importantly, the role of grid cells is not confined to active navigation [144]. A growing body of evidence implicates the MEC and its grid network in trajectory replay, and spatial planning [113,163]. Grid-like activity has been observed in humans during imagined navigation tasks, providing evidence that grid cells participate in mental simulation and prospective planning even in the absence of physical movement [43,164]. Grid cells in the MEC and place cells in the HPC exhibit predictive firing, typically shifting their activity a few hundred milliseconds ahead of the animal’s actual position [165,166]. This suggests that the spatial metric provided by grid cells is repurposed for internal cognitive operations, such as planning future paths or recalling past trajectories [43,164]. These predictive representations are organized by theta oscillations and expressed during the trough phase of the cycle, supporting the idea of a temporally structured predictive map [165,166]. The phenomenon of theta phase precession, wherein spatial information is encoded as a function of oscillatory phase, complements this view by showing how sequences from past to future positions may be represented within each theta cycle [167,168]. The discovery of predictive grid cells points to a potential role for the MEC in supporting the formation of predictive cognitive maps. However, much remains to be clarified regarding the processing of predictive spatial information along the HPC-EC axis [166]. Replay phenomena, in which previously experienced movement patterns are reactivated during rest, further support the hypothesis that grid cells are involved in retrieving and reinforcing spatial memory [113,163]. During sleep, the correlation structure between intramodular grid cells is preserved in a manner predicted by their spatial phase relationships during wakefulness [169,170]. This stability, which is arousal-state-independent, suggests that the underlying network dynamics responsible for the grid pattern persist even in the absence of external input. Correlation strength during sleep is highest between intramodular cell pairs with similar phases and declines for intermodular pairs, whose relationships tend to degrade during slow-wave sleep [169]. The persistence of this fine-grained correlation structure during offline states implies a role for sleep in the maintenance or consolidation of the spatial metric code, reinforcing the idea that grid cell dynamics underlie a fundamental cognitive map used during both online navigation and offline processing [144,169,170]. Notably, such replay in the MEC can occur independently of HPC sharp-wave ripples, indicating that the EC possesses intrinsic mechanisms for memory processing [159,169].

### 3.2. Non-Grid Spatial Cells

Alongside grid cells, the MEC houses a diverse array of non-grid spatial cells that encode complementary aspects of navigation [147]. These include border cells that signal proximity to environmental boundaries, HD cells that encode orientation, and speed cells that represent velocity [63,65,147]. Object-vector (OV) cells encode distances and directions to objects, and other non-grid spatial (NGS) cells contribute additional spatial representations through non-periodic, heterogeneous firing patterns [65,171]. Notably, some neurons exhibit hybrid properties, such as conjunctive cells, which integrate phase-modulated spatial coding with direction selectivity [50,63]. These cells often display strong phase-locking to the theta rhythm and participate in functional circuits that bridge pure grid and HD cells, contributing to a more integrated spatial representation [50,172].

Speed cells are a specialized population of the MEC that further support navigation [165,173,174]. Originally identified as constituting approximately 15% of MEC neurons, these cells exhibit a linear, positive correlation with running speed and encode this information independently of visual input [165,175]. Although initially thought to be distinct from other spatially tuned cell types, subsequent analyses suggest a broader and more heterogeneous population of speed-modulated neurons, with estimates ranging from 30% to 80% of MEC cells showing various forms of speed tuning [165,176]. While many of these exhibit weak modulation and often encode speed in conjunction with other variables such as position or direction, the dedicated linear subset appears particularly effective for decoding instantaneous velocity [176]. A substantial portion of speed cells apparently belongs to the class of fast-spiking interneurons, and approximately 83% of HPC-projecting fast-spiking cells (putative PV^+^ neurons) in MECII and III are modulated by the animal’s speed and project to the HPC, potentially contributing to self-location representation [175].

In parallel, egocentric spatial representations, previously thought to be absent or minimal in the EC, have now been identified within both the MEC and the LEC. These include cells tuned to egocentric bearing and distance to environmental features such as the center or boundaries of the enclosure, often located in the deeper layers of the MEC [127,149]. The firing of these egocentric cells is consistent across different contexts and remains anchored to local environmental structure, resembling similar coding properties observed in the POR cortex, which provides upstream input to MEC [113,127]. The LEC, traditionally associated with non-spatial object identity processing, is increasingly recognized as encoding spatial aspects of the local environment, particularly those that are object- or proximity-related [47,76]. Thus, egocentric representations in the EC likely rely on both self-motion signals and proximal environmental cues [113,127].

Anatomically, while some non-grid cell types show tendencies toward spatial clustering, only grid cells consistently form stable groupings of three or more anatomically adjacent cells [10]. Non-grid cells are more likely to be interspersed in a “salt-and-pepper” distribution, which may reflect different network demands or input sources [157,176]. This less spatially regimented organization could suggest either a reliance on upstream inputs or an embedding within broader, more distributed functional networks.

### 3.3. Path Integration

Through continuous integration of directional and velocity signals, animals are capable of estimating their current location and orientation in an allocentric reference frame, effectively computing a vector that points back to the starting point [177,178]. This capacity is especially vital for homing behaviors in environments where external landmarks are scarce or unreliable [129,179]. This process is called path integration (PI), also referred to as dead reckoning, and is based primarily on idiothetic information, internally generated cues arising from self-motion, such as proprioceptive, vestibular, and motor signals, with the integration of allothetic cues that derive from the external environment, including both distal and proximal landmarks [129,180]. Central to this function is the MEC, which is widely recognized as a core component of the neural substrate underlying self-referenced navigation and has been robustly implicated in PI, serving as the site where idiothetic and allothetic signals are integrated to maintain and update spatial representations [181,182,183,184]. Grid cells are central to this function, with models of PI often using their periodic firing as a substrate for updating position in the absence of visual landmarks [178,182]. While the MEC and LEC both process allothetic inputs, the MEC is particularly tuned to spatial cues from distal landmarks and multiple sensory modalities [47,185]. This integrative capacity allows the animal to switch between or combine path integration and map-based strategies, depending on environmental stability or sensory availability [148,186]. For instance, in environments devoid of stable landmarks or in total darkness, path integration becomes the dominant strategy [186,187]. This dynamic interaction is thought to be mediated, in part, by excitatory projections from the POR to the MEC, which provides a continuous stream of spatially relevant sensory information, and may contribute to the EC’s ability to maintain consistent spatial representations across contexts, while inhibitory projections from the PER to the LEC suggest a mechanism for selective gating of object-related (and potentially proximal) cues [39,47,113].

Experimental evidence further substantiates the MEC’s role in path integration. Lesions in the MEC degrade performance in navigational tasks requiring self-motion-based localization, and experimental disruption of the grid code selectively impairs the accuracy of path integration without abolishing other MEC signals [186,188,189]. These findings suggest that grid cell activity in particular is not merely a product of the movement but a necessary element of navigational computation [185,188].

Importantly, PI is inherently noisy in self-motion signals, with cumulative error increasing over time in the absence of external references [179,186]. PI accuracy declines steeply with increased distance, and systematic drifts can emerge, particularly under conditions of low velocity input [186,188]. To counteract these degradations, sensory landmarks serve to recalibrate the internal spatial representation [179,189,190]. Cue cells, a recently characterized neuronal population in the MEC, respond reliably to prominent spatial cues, exhibiting repeated and localized firing fields near visual features, and may act as anchors, adjusting internal estimates and maintaining consistency across navigation, offering a more precise mechanism for integrating visual information into the MEC’s spatial code [190]. Although distinct from grid and border cells, some cue cells exhibit conjunctive properties, suggesting a heterogeneous coding strategy for environmental features [113,191]. Border cells, which fire at environmental boundaries, further anchor spatial representations and contribute to recalibration mechanisms [187,192]. These external inputs, when processed through the MEC, allow for dynamic adjustment of internal representations, correcting drift and enhancing accuracy [113,185]. Beyond immediate correction, experience with an environment refines grid representations further [193]. Initially fragmented or locally anchored grid patterns, shaped by local cues, can coalesce into a globally coherent map with repeated exposure and navigation, particularly following loop closures [113,185]. This transformation reflects a self-corrective process whereby the entorhinal system integrates local sensory and motion-derived inputs over time to produce a unified spatial representation [39,185,194].

PI also relies critically on speed information, which is encoded in both the MEC and CA1 [113,181]. Within the MEC, speed cells exhibit firing rates that scale linearly with locomotion velocity, providing a direct representation of self-motion, enabling continuous updating of spatial position [181,193]. While grid cells are modulated by speed, dedicated speed cells offer a more consistent readout for velocity decoding [165,181].

In CA1, speed-tuned neurons, primarily inhibitory interneurons, also show firing patterns correlated with movement speed, though via a distinct encoding scheme [165,178]. The EC conveys speed signals to CA1 through both direct and trisynaptic pathways: ECII neurons encode speed prospectively, while ECIII neurons do so retrospectively, delivering temporally complementary inputs that may support the integration of ongoing and previously experienced movement information across different behavioral timescales [113,165,179,181].

Importantly, MEC speed signals adapt alongside changes in grid scale when the environment is deformed, revealing a co-rescaling mechanism that reflects the animal’s perception of movement through space [113]. This challenges the earlier view of speed cells as context-invariant and supports a more integrated model where internally generated velocity signals are dynamically modulated by external environmental cues. Although debate remains about the temporal resolution of speed coding, namely whether speed cells reflect moment-to-moment velocity or broader behavioral states, their interaction with theta oscillations likely provides a rhythmic scaffold for encoding self-motion in a spatially and temporally structured manner [113,185].

Theta oscillatory activity, within the 4–12 Hz theta range, plays a central role in coordinating the temporal alignment of multiple self-motion-related signals and is thought to enable temporal coding strategies such as phase precession, particularly in grid and stellate cells of the MEC [178,186]. Stellate cells, with their intrinsic membrane potential oscillations, are considered critical for spatial learning and may facilitate coordination of speed-modulated directional input through phase modulation [100,154]. Loss of HPC theta activity leads to spatial memory deficits in rodents, while in humans, theta activity correlates with movement speed and orientation during virtual navigation [113,181,189].

Theta oscillations facilitate the organization of information flow within the spatial navigation network by temporally structuring activity across the parahippocampal formation [195]. This rhythmicity is thought to segment neural processing into discrete time windows, enabling the reception and filtering of sensory inputs and supporting the integration of new stimuli into ongoing cognitive processes [185]. Experimental evidence demonstrates that vestibular inputs are necessary for maintaining theta oscillations in the EC [196]. Disruption of vestibular function abolishes velocity-modulated theta activity in the EC and destabilizes the spatial periodicity of grid cells in the MEC, underscoring the critical role of theta in integrating self-motion cues and sustaining spatial representations [178,197]. Beyond internal cue processing, theta rhythm is also implicated in integrating PI with allothetic navigation [113]. It provides a temporal scaffold for spatial coding in both MEC and HPC, wherein spatially selective neurons, such as grid cells and place cells, fire at specific phases of the theta cycle [113,195]. A particularly salient phenomenon, theta phase precession, describes how neuronal spikes occur at progressively earlier phases of the theta cycle as an animal crosses a firing field. This mechanism integrates temporal sequences into a coherent spatial-temporal representation, supporting navigation [113,195,198]. Reduced theta phase precession, observed in models of early AD, correlates with deficits in distance estimation and grid cell function, emphasizing the importance of precise theta-speed coupling for effective PI [181,182,196].

Crucially, theta rhythms do not operate in isolation. They interact with gamma oscillations (30–130 Hz), another class of rhythmic activity that plays a vital role in coordinating neuronal activity across the cortex [185,199]. Within the EC-HPC circuit, gamma oscillations organize spike timing among distributed neuronal assemblies and facilitate precise inter-regional communication. Theta-gamma coupling allows upstream and downstream neurons to synchronize their activity within defined temporal windows, enhancing information flow that supports the encoding and retrieval of spatial representations along specific pathways, such as MEC–DG–CA3 [179,188].

Apart from HD cells not strictly theta-dependent, the interplay between non-grid cells and theta oscillations is less uniformly characterized than for grid cells [113]. Nonetheless, several non-grid populations display theta modulation. Conjunctive cells, by contrast, show phase-specific theta firing and interneurons, including those expressing SST^+^ or PV^+^, differentially influence the rhythmicity and spatial selectivity of grid and non-grid populations. Interestingly, in the absence of visual cues, MEC population-level synchrony may compensate for diminished single-cell rhythmicity, stabilizing network activity [113,196,199].

Disruptions in these integrative processes are evident in models of neurodegenerative diseases such as tauopathy and AD [181,196,200]. Mouse models of AD have shown that deficits in locomotor speed encoding and theta-phase coupling in MEC neurons precede broader cognitive decline [196,201]. The accumulation of phosphorylated tau in the MEC impairs grid cell regularity and impaired distance estimation mirrors the navigational deficits observed behaviorally. Notably, PI impairments often precede overt memory symptoms, suggesting that PI tasks may serve as early diagnostic tools for identifying individuals at risk of AD or mild cognitive impairment (MCI) [196]. Such findings highlight the vulnerability of MEC-mediated PI circuits and underscore their foundational role in maintaining spatial cognition.

In summary, the HPC constructs a topological cognitive map thanks to the interactions with the MEC and LEC, providing a stable representation of space, also showing substantial context-specific modulation [39,202,203]. During loop closures, moments when an animal returns to a previously visited location, errors accumulated via PI can be corrected through sensory feedback [185,204]. The HPC then projects these corrections back to the MEC, potentially via sharp wave ripples, to update grid and HD cell states, enabling globally coherent spatial representations. This iterative loop between the EC and HPC supports the construction of a globally coherent spatial map [185,205]. Collectively, these findings highlight PI as a fundamental mechanism for flexible navigation and identify the EC, particularly the MEC, as a core neural substrate supporting the continuous integration and correction of self-motion and sensory information during spatial behavior.

## 4. Temporal Representation

Beyond its role in memory and spatial navigation, the EC also contributes critically to the organization of experiences across time, enabling the association of temporally discontinuous events into coherent representations. Temporal representation refers to the neural mechanisms supporting the encoding and processing of information about time, including not only the organization of events in memory, but also the perception and tracking of elapsed time and temporal intervals during ongoing experience and behavior [19,23]. The EC and HPC together form a central hub for this aspect. Within this network, the MECIII and MECII emerge as pivotal regions. Through the temporoammonic route, MECIII neurons provide direct, monosynaptic excitatory projections to CA1 pyramidal neurons, crucial for temporal association learning (TAL), a process that links non-contiguous events across time [19,23,25].

### 4.1. MEC’s Role in Temporal Representation

In paradigms such as trace fear conditioning (TFC), where a conditioned stimulus is followed by a temporal gap (typically 20–30 s) before the unconditioned stimulus, disruption of MECIII activity impairs the animal’s ability to form such temporal links, while spatial memory remains largely unaffected [23,25]. As such, mice with inhibited MECIII cells showed deficits in TAL during TFC tests, while their performance in normal CFC remained unaffected. These deficits are prevented when the trisynpatic pathway, particularly via Re^+^ cells, is impaired [5]. Conversely, optogenetic stimulation of MECIII cells has been reported to increase TFC test performance [52].

**Figure 3:**
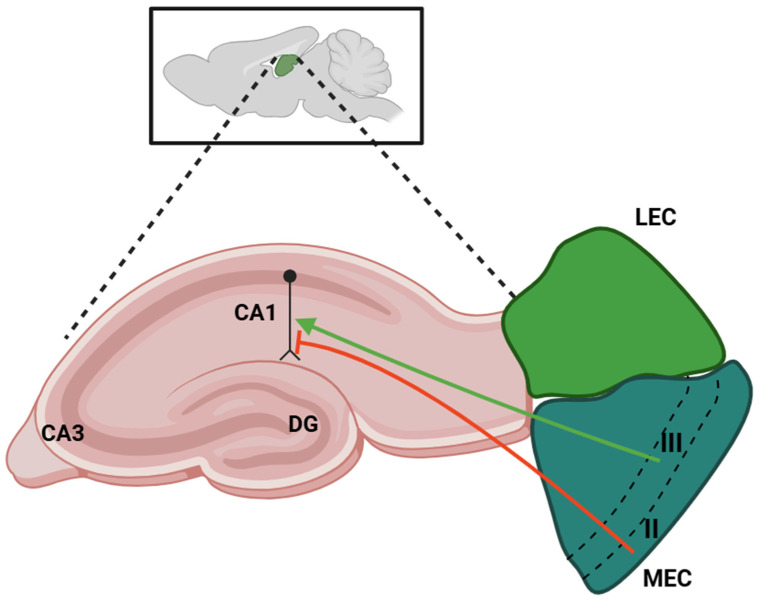
Temporal gating circuits within the entorhinal–hippocampal network. The MECIII→CA1 temporoammonic pathway (green arrow) provides excitatory temporal association signals necessary for linking temporally discontinuous events. Through persistent firing and temporally predictive activity, MECIII neurons maintain cue-related information across delay periods and transmit it directly to CA1 pyramidal neurons, thereby supporting temporal association learning and episodic binding. In contrast, MECII island cells indirectly regulate this process through feedforward inhibition (red pathway) by activating SL interneurons, which modulate the strength and timing of temporoammonic inputs onto distal CA1 dendrites. Together, these complementary excitatory and inhibitory mechanisms coordinate temporal precision and gating within the EC–hippocampal circuitry. DG: dentate gyrus; CA3: cornu ammonis 3; CA1: cornu ammonis 1; LEC: lateral entorhinal cortex; MEC: medial entorhinal cortex. Dotted lines refer to the zoom-in of the regions of interest. Roman numbers refer to LEC and MEC Layers.

Possible neural mechanisms underlying MECIII’s role include spontaneous persistent firing, which has been observed in MECIII in vivo during slow-wave sleep and potentially during waking behavior [206]. This persistent activity could serve as a “temporal buffer” to retain information across the temporal gap between events, thereby enabling CA1 to associate these signals [23,105]. Yet, this excitatory input from MECIII does not operate in isolation. It is subject to a sophisticated regulatory mechanism mediated by a separate but interconnected circuit involving MECII Island cells [23]. These Cal/Wfs1^+^ pyramidal excitatory neurons are organized in distinct modules and project to GABAergic interneurons in the SL of CA1 [23,102]. The SL lies directly below the stratum moleculare (SM), where the axonal terminals of MECIII neurons synapse onto the distal dendrites of CA1 pyramidal cells [23,25]. This precise laminar organization permits a feedforward inhibitory mechanism in which Island cell activation excites SL interneurons, which in turn suppress MECIII input to CA1 pyramidal cells (Figure 3) [23,25]. As a result, when Island cells are active during the critical stimulus-free interval of TFC, the gating they provide impairs the integration of MECIII signals, thus inhibiting temporal association memory formation [4,23,26]. Conversely, silencing Island cells removes this gating, enhancing MECIII signal efficacy and facilitating temporal memory acquisition. This bidirectional modulation of TFC learning is further highlighted by optogenetic manipulation of Island cell projections to CA1 during trace intervals where optogenetic activation impairs TFC learning by suppressing MECIII input, whereas the inhibition enhances TLA by facilitating MECIII signal integration. This interplay ensures that only appropriately timed signals from MECIII are transmitted effectively to CA1, thereby enabling precise temporal association [4,23].

The MEC inhibitory microcircuits further refine this integration by involving SST^+^ GABAergic interneurons that preferentially target pyramidal neurons in layers III-V, and PV^+^ GABAergic interneurons, that project directly to the SLM of CA1 [4,23,95]. In particular, these PV^+^ projections are largely arising from fast-spiking PV^+^ cells. While the specific HPC targets of these PV^+^ projections are not fully delineated, their location in SLM suggests they modulate dendritic integration zones of CA1 pyramidal neurons or local interneurons. This dual modulation (direct GABAergic inhibition and indirect inhibition via excitatory Cal/Wfs1^+^ projections) supports a multifaceted regulatory framework for controlling the timing and integration of temporally modulated EC inputs in CA1 (Figure 3) [4,25,53,95].

The MEC function of representing elapsed time is encoded via the sequential activity of time-sensitive neurons, commonly referred to as “time cells”, that exhibit firing patterns that are selectively tuned to specific moments within a temporal interval and collectively form a sequential activity pattern that effectively tiles or spans the entire duration of the timing period in a manner reminiscent of a clock [92,207,208]. These time cells provide a neural substrate for tracking the passage of time within a given context, and their activity is integral to the HPC time cell sequences believed to underline the temporal structure of episodic memory [208,209]. Importantly, this temporal representation in MEC is not static; rather, it is modulated by learning. Specifically, as animals learn to differentiate between different temporal contexts or structures of trials, distinct patterns of time cell activity emerge [92,208]. These unique patterns are proposed to form temporal maps of distinct experiences, enabling the animal to flexibly learn and navigate complex temporal contingencies. MEC’s role is particularly important for flexibility and updating temporal context, rather than simply timing fixed intervals [44,208].

The transient inactivation or lesions of the MEC disrupt this finely tuned temporal coding, with particularly pronounced effects on tasks that demand the discrimination of longer temporal intervals [19,92]. For instance, in a time duration discrimination (TDD) task rats are required to differentiate between 10 s and 20 s delay intervals. Once a performance level of 90% was reached, rats received excitotoxic MEC lesions or sham lesions. MEC-lesioned animals displayed significant impairments in discriminating the longer interval [19]. While their performance on 10 s trials improved modestly with continued training, these animals failed to recover preoperative levels of accuracy on 20 s trials, even after 50 days of testing. This dissociation suggests a delay-dependent disruption attributable to MEC damage, implicating the MEC as particularly necessary for estimating time spans [19]. In particular, when longer delays (i.e., 60 s) vs. short 10 s delays have been tested, the MEC has been found crucial for delays longer than 10 s. Simultaneously, neural recordings reveal that HPC circuits, which integrate EC inputs, can represent elapsed time through sequential firing patterns across both 10 s and 60 s intervals. This suggests that while the capacity to represent intervals exists through these durations, the EC, particularly MEC, may play a more prominent role in supporting the behavioral utilization or processing of information over longer temporal scales that potentially exceed immediate WM capabilities [19,210].

Complementing these findings, MEC exhibits minute-scale oscillatory sequences (ultraslow and periodic activity patterns extending across tens of seconds to minutes) that are absent in adjacent brain areas [211]. Though not directly linked to the TDD’s 10- vs. 20 s trials, the timescales of these oscillations align with those temporal durations where MEC function is most critical, such as during TFC or long-delay discrimination tasks [25]. It is hypothesized that such ultraslow oscillations may serve to couple neurons across extended timescales, potentially supporting the formation of coherent temporal frameworks necessary for episodic memory. This organizing principle may contribute the MEC’s ability to scaffold timelines upon which temporally structured experiences are encoded and retrieved. These dynamics have been observed in MEC neuronal activity while animals engaged in behavior such as running at free pace in darkness on a rotating wheel, notably without changes in location, running direction, or scheduled rewards [62,208,211]. The sequences were found to involve nearly the entire cell population and persisted even during epochs of immobility. Importantly, similar sequences were not detected in neighboring brain regions like the PaS or visual cortex, suggesting a degree of specificity to the MEC [211]. Moreover, the MEC neurons temporal representations become increasingly differentiated with experience and exhibit characteristic disruptions in cases of errors, further supporting a role for MEC activity in acquiring and maintaining temporal structure through an error-correction mechanism [92,208].

Time-varying and non-varying properties of neurons within CA1 and CA2 are shaped by these MEC entorhinal inputs [139,212]. Although intrinsic evidence of persistent time-varying activity within CA1 or CA2 is limited, CA1 cells exhibit dynamic context-sensitive firing, suggesting they may inherit time-varying signals from upstream sources [97,139,210]. Thus, the HPC receives a confluence of stable spatial and dynamic temporal signals, with CA1 integrating these composite inputs [97,210].

#### Temporal Representation in the MEC: Pathological Aspects

The relevance of MEC temporal progression is further underscored by its vulnerability to age-related and pathological alterations [3,213]. In models of AD and TLE, selective impairments in MEC circuits, particularly in MECII stellate cells and MECIII excitatory neurons, disrupt the integrity of temporal coding and synchronization, correlating with the emergence of memory deficits [102,115,214]. Accumulation of phosphorylated tau protein in Cal/Wfs1^+^ cells in MECII has been observed in early AD models, followed by propagation to CA1 [25]. More detailed studies in the 3xTg mouse model of AD pathology have tracked changes in intrinsic excitability and synaptic activity in distinct excitatory cell types, from both MECII and MECIII, at early pre-symptomatic (3 months) and late post-symptomatic (10 months) time points. At the early stage, MECII stellate and pyramidal cells showed an increase intrinsic excitability. This hyperexcitability was counteracted by a shift in synaptic inputs towards relatively more inhibition and less excitation, suggesting homeostatic control. In contrast, MECIII neurons showed the opposite effect with an intrinsic reduced excitability. By a late stage, while MECII pyramidal and MECIII excitability largely normalized, MECII stellate cells remained intrinsically hyperexcitable, suggesting a breakdown in homeostatic mechanisms specifically in this cell type. This increased excitability in MECII stellate cells at the post-symptomatic stage is suggested to contribute to the emergence of memory deficits in AD [101]. While not directly affecting temporal representation, the hyperactivity of MECII stellate cells disrupts the microcircuitry that regulates and supports the temporal function of MECIII, thereby contributing to deficits in temporal processing observed in AD [92,101].

In models of TLE, memory deficits are associated with disruptions in MEC circuits [106,115]. Severe, late-onset impairments are linked to disrupted synchrony of MEC circuits. Specifically, excitatory neurons in MECIII, active near the trough of theta oscillations, is specifically disrupted in epileptic mice, and this disruption corresponds with the severity of memory impairments. While inhibitory neurons in MECII and MECIII show minimal changes in theta phase locking, MECII inhibitory cells show an increase in firing rate in older epileptic mice. There is decreased theta coherence within the MEC and between the MEC and HPC that emerges between 3 and 8 weeks after epilepsy induction [106]. Given the crucial role of the MECIII-CA1 pathway in temporal association formation, the preferential neuronal loss in MECIII, along with the selective disruption of MECIII neurons and their impaired temporal firing precision, observed in TLE, significantly impairs the temporal coding processes, thereby contributing to the temporal representation deficits characteristic of the disorder [4,5,106].

### 4.2. LEC’s Role in Temporal Representation

An additional layer of complexity in temporal encoding is introduced by the LEC, a region historically linked with object-related information but increasingly recognized for its contributions to temporal representation [32,122]. Many LEC neurons exhibit temporal modulation of firing rates, a phenomenon known as rate remapping, at a strength surpassing that seen in MEC, CA1, or CA3. LEC firing patterns reflect not only elapsed time within a trial but also the broader temporal context of experience, extending from seconds to hours [25,32,122]. Importantly, LEC neurons maintain temporal modulation across different experimental sessions and synchronize with mPFC and HPC activity during temporal association memory tasks [122]. This temporal modulation suggests that LEC neurons multiplex spatial (as seen in the previous chapter) and temporal data, potentially coding for temporal context and allowing for flexible integration of these aspects [122,215].

Importantly, LEC neurons not only respond to sensory stimuli but also maintain information about prior events through both phasic and tonic modes of activity [36,122,216,217]. This dual coding strategy allows for continuous signaling of environmental events, even in the absence of immediate stimuli, thereby supporting the prospective retrieval of past events [216,217]. The abrupt shifts in LEC firing patterns upon re-entry into familiar environments underscore the sensitivity of these neurons to the sensory history of those environments, suggesting a mechanism for recalling contextualized experiences based on current sensory input [122,216]. Such context-dependent recall is foundational for TAL, which necessitates the binding of temporally distant events. LEC neurons’ tonic firing during inter-stimulus intervals provides a sustained signal that bridges gaps between events, offering a potential neural substrate for linking cause and effect across time [4,23,216]. Collectively, these results suggest that the LEC may signal not only what is currently happening but also what had happened previously in a specific environment [216].

Altogether, these findings highlight the central role of the EC, through complementary contributions of MEC and LEC, in generating temporally structured signals via distinct excitatory circuits. While MEC supports the encoding of elapsed time through sequential neuronal dynamics, LEC may contribute time-encoded information linked to experience and context [36,217,218]. These temporally organized outputs provide the HPC with the necessary structure to integrate and map events across time [23,92,218]. Overall, these findings suggest that the LEC contributes to temporal representation by linking sensory experiences to their temporal context, thereby supporting the formation of temporally structured episodic memories. Furthermore, the fact that these mechanisms are selectively altered in pathological conditions further underscores their fundamental role in temporal representation.

## 5. Conclusions

The EC is a structurally conserved and functionally pivotal region within the medial temporal lobe, playing a fundamental role in bridging neocortical sensory inputs and HPC computations. Across this review, we have outlined in detail how the EC’s anatomical organization, cytoarchitecture, and connectivity support a rich array of cognitive functions, particularly those related to memory, navigation, and temporal representation.

The EC, long conceptualized as the gateway to the hippocampal formation, has traditionally been portrayed through a dichotomy: the MEC as the processor of spatial, metric, and self-motion cues, and the LEC as the conduit for non-spatial, sensory, and contextual information. While this framework has guided decades of research, it is insufficient to capture the complex functional architecture of the EC. The MEC plays a key role in allocentric spatial processing, path integration, and temporal sequence learning. Conversely, the LEC, once considered functionally marginal, has increasingly been recognized for its role in encoding object identity, contextual features, and temporal aspects of episodic memory. Although they exhibit well-characterized specializations, MEC and LEC do not operate as independent modules. Their dynamic and integrated functions jointly shape hippocampal coding of space, time and experience. This integrated view suggests that memory, navigation and temporal representation depend less on parallel, insulated pathways and more on a flexible exchange of allocentric and egocentric, spatial and non-spatial, and metric and associative cues. Importantly, beyond representing where events occur and what they contain, EC circuits appear critical for binding experiences across time, enabling temporally discontinuous events to be associated into coherent episodic representations.

Importantly, these computations may reflect broader principles of cognitive map formation and predictive coding, whereby the EC dynamically integrates internal representations with incoming sensory information to generate flexible models of ongoing experience. In this context, spatial and temporal coding may not represent fundamentally distinct operations, but rather complementary dimensions of a unified computational framework supporting episodic organization and behavioral prediction. Within this framework, temporal representation is not limited to interval timing, but may provide the organizational scaffold through which sequential experiences are linked, updated, and recalled across behaviorally relevant timescales.

Nonetheless, several unresolved questions reveal the complexity of this integrative system. One persistent gap concerns the precise circuit mechanisms through which MEC- and LEC-derived information interact; although cross-modulation is well documented, the underlying principles, particularly the respective roles of excitation and inhibition, remain elusive. Similarly, the functional relevance of EC layers remains poorly characterized, despite their proposed involvement in long-range interactions that stabilize and transform memory traces over time. Moreover, while oscillatory coordination between EC, hippocampus, and prefrontal cortex is widely observed, the causal contribution of these rhythms to temporal association, predictive coding, and flexible navigation is not yet fully understood. An additional unresolved issue concerns how EC computations are modulated by behavioral state, attentional demands, motivational salience, and task structure, as these factors likely shape the balance between internally generated representations and externally driven sensory information.

These gaps are further underscored by the EC’s pronounced vulnerability in disorders such as AD and TLE. Selective degeneration of specific layers and cell types suggests that the integrative operations of the EC may be particularly susceptible to disruption. Understanding why MEC grid cells, LEC fan cells, or deep output layers exhibit differential pathology may illuminate not only disease progression but also the computational demands placed on the EC.

While the reviewed studies provide valuable insights, several methodological limitations should be acknowledged. First, there is considerable heterogeneity across rodent studies in terms of animal strain, species, sex, age, genetic background, and experimental disease models, as well as differences in housing conditions and sample size, all of which may reduce the reproducibility and generalizability of the findings. In addition, the studies employed diverse experimental paradigms, making direct comparisons across results difficult. Moreover, although several findings suggest promising therapeutic implications, most evidence currently derives from preliminary rodent models and therefore should be interpreted cautiously with respect to direct clinical translation. Finally, differences in outcome measures and reporting standards may have introduced additional bias and contributed to inconsistencies within the literature.

Future research should therefore move beyond the dichotomy of a “spatial MEC” and “non-spatial LEC” toward an understanding of how these subregions cooperate under different cognitive demands. Combining high-resolution circuit dissection with behavioral paradigms will be essential for determining how MEC–LEC interactions contribute to memory, predictive coding, and flexible navigation. Integrating these approaches with computational frameworks may further clarify how the EC transforms multimodal sensory information into generalized representations of space, time, and context that can flexibly guide behavior. Particularly important will be understanding how EC microcircuits support the temporal binding of events, allowing experiences separated in time to be integrated into unified episodic memories.

Taken together, the EC is not merely a conduit for HPC inputs and outputs, but a dynamic and integrative structure essential for organizing experience across space, time, and context. Its internal architecture, input and output specificity enable it to contribute uniquely to the construction of episodic, spatial and working memory, spatial navigation, and temporal representation. Importantly, converging evidence from rodent circuit studies, human neuroimaging, and intracranial electrophysiology strongly reinforces the view that the EC-HPC system represents a conserved neural substrate for organizing and binding experiences across behaviorally relevant timescales. In particular, the EC appears uniquely positioned to bridge temporally discontinuous events, providing neural mechanisms through which experiences can be sequentially organized and bound into coherent episodic structures. As our understanding of its microcircuitry, connectivity, and modulatory systems deepens, the EC stands as a critical focal point for unraveling the neural basis of cognition and for developing interventions targeting memory dysfunction in neurological disease.

## Data Availability

No new data were created or analyzed in this study.

## Abbreviations

The following abbreviations are used in this manuscript:

AD	Alzheimer’s disease
alEC	Anterolateral entorhinal cortex (human homolog of rodent LEC)
BLA	Basolateral amygdala
CA1, CA2, CA3	Cornu Ammonis hippocampal subfields 1, 2 and 3
Cal/Wsf1^+^ cells	Calbindin- and Wolfram syndrome 1-positive pyramidal neurons
CFC	Contextual fear conditioning
DBS	Deep brain stimulation
DG	Dentate gyrus
DMP	Delayed matching-to-place task
DNMP	Delayed non-match to place task
DNMO	Delayed non-match to sample based on odors
EC	Entorhinal cortex
EC–HPC circuit	Entorhinal cortex–hippocampal circuit
fMRI	Functional magnetic resonance imaging
GABA	Gamma-aminobutyric acid
HD cells	Head direction cells
HPC	Hippocampus
IEGs	Immediate early genes
LEC	Lateral entorhinal cortex
LECII/LECIII	Layer II/Layer III of the lateral entorhinal cortex
LPP	Lateral perforant path
LTP	Long-term potentiation
MEC	Medial entorhinal cortex
MECII/MECIII	Layer II/Layer III of the medial entorhinal cortex
mPFC	Medial prefrontal cortex
OV cells	Object-vector cells
PaS	Parasubiculum
PER	Perirhinal cortex
PI	Path integration
pmEC	Posteromedial entorhinal cortex (human homolog of rodent MEC)
PNNs	Perineuronal nets
POR	Postrhinal cortex
PrS	Presubiculum
PV^+^ cells	Parvalbumin-positive interneurons
Re^+^ cells	Reelin-positive neurons
SL/SLM	Stratum lacunosum/stratum lacunosum-moleculare
TBS	Theta-burst stimulation
TLE	Temporal lobe epilepsy
WM	Working memory
